# Potential of *LP* as a Biocontrol Agent for *Vibriosis* in Abalone Farming

**DOI:** 10.3390/microorganisms13071554

**Published:** 2025-07-02

**Authors:** Ling Ke, Chenyu Huang, Song Peng, Mengshi Zhao, Fengqiang Lin, Zhaolong Li

**Affiliations:** 1 The Research Institute of Biotechnology, Fujian Academy of Agricultural Sciences, Fuzhou 350013, China; keling@faas.cn; 2 Institute of Animal Husbandry and Veterinary Medicine, Fujian Academy of Agricultural Sciences, Fuzhou 350013, China; nimoy88@163.com (C.H.); pengsong@faas.cn (S.P.); 13375001253@163.com (M.Z.); linfengqiang@faas.cn (F.L.)

**Keywords:** *LP*, abalone, vibrio, inhibition

## Abstract

*Vibrio* species are among the primary pathogenic bacteria affecting abalone aquaculture, posing significant threats to farming practices. Current clinical control predominantly relies on antibiotics, which can result in antibiotic residues in both abalone and the surrounding marine environments. *Lactobacillus plantarum* (*LP*) has been shown to release bioactive antagonistic substances and exhibits potent inhibitory effects against marine pathogenic bacteria. This study aimed to screen and characterize the probiotic properties of *LP* strains isolated from rice wine lees to develop a novel biocontrol strategy against *Vibriosis* in abalone. The methods employed included selective media cultivation, streak plate isolation, and single-colony purification for strain screening, followed by Gram staining, 16S rDNA sequencing, and phylogenetic tree construction using MEGA11 for identification. The resilience, antimicrobial activity, and in vivo antagonistic efficacy of the strains were evaluated through stress tolerance assays, agar diffusion tests, and animal experiments. The results demonstrated the successful isolation and purification of four *LP* strains (NDMJ-1 to NDMJ-4). Phylogenetic analysis revealed closer genetic relationships between NDMJ-3 and NDMJ-4, while NDMJ-1 and NDMJ-2 were found to be more distantly related. All strains exhibited γ-hemolytic activity, bile salt tolerance (0.3–3.0%), and resistance to both acid (pH 2.5) and alkali (pH 8.5), although they were temperature sensitive (inactivated above 45 °C). The strains showed susceptibility to most of the 20 tested antibiotics, with marked variations in hydrophobicity (1.91–93.15%) and auto-aggregation (13.29–60.63%). In vitro antibacterial assays revealed that cell-free supernatants of the strains significantly inhibited *Vibrio* parahaemolyticus, V. alginolyticus, and V. natriegens, with NDMJ-4 displaying the strongest inhibitory activity. In vivo experiments confirmed that NDMJ-4 significantly reduced mortality in abalone infected with V. parahaemolyticus. In conclusion, the *LP* strains isolated from rice wine lees (NDMJ-1 to NDMJ-4) possess robust stress resistance, adhesion capabilities, and broad antibiotic susceptibility. Their metabolites exhibit significant inhibition against abalone-pathogenic Vibrios, particularly NDMJ-4, which demonstrates exceptional potential as a candidate strain for developing eco-friendly biocontrol agents against *Vibriosis* in abalone aquaculture.

## 1. Introduction

Abalone aquaculture, a cornerstone of global marine fisheries, plays a pivotal role in driving economic development in coastal regions. However, the health and productivity of farmed abalone are severely threatened by pathogenic microorganisms prevalent in marine environments, with *Vibrio spp*. emerging as one of the most detrimental bacterial genera. Pathogenic *Vibrio* species, including *V. parahaemolyticus*, *V. alginolyticus*, and *V. natriegens*, invade abalone through contaminated water or feed, colonize the intestinal tract, and disrupt gut microbiota homeostasis. This dysbiosis leads to suppressed growth performance manifested as reduced feeding, stunted growth, and weight loss, and may trigger systemic immunosuppression and infections, resulting in mass mortality events that jeopardize both economic returns and ecological security in aquaculture systems [1].

Antibiotics remain the primary strategy for controlling Vibrio-related diseases in abalone farming. However, their overuse has precipitated critical challenges: (1) prolonged antibiotic use has accelerated the development of pathogen resistance, compelling farmers to increase dosages or switch drugs, thereby escalating production costs; (2) antibiotic residues accumulate in abalone tissues and surrounding marine environments, posing risks to human health via the food chain; and (3) drug dispersion disrupts marine ecosystems by promoting the horizontal transfer of resistance genes among environmental microbes and inducing toxic effects on non-target organisms. Consequently, there is an urgent need to develop eco-friendly, sustainable alternatives to antibiotics to ensure the long-term viability of abalone aquaculture and marine ecosystem stability.

Probiotics, particularly *LP*, have gained attention as safe and multifunctional agents for disease prevention in aquaculture. *LP* enhances host health through multiple mechanisms: its metabolites, such as organic acids, exopolysaccharides (EPS), and bacteriocins, directly inhibit pathogen growth and competitively exclude pathogenic colonization. For instance, organic acids (e.g., lactic acid and acetic acid) secreted by *LP* lower intestinal pH, destabilizing the cell membrane integrity of *Vibrio* spp. and impairing their energy metabolism. Bacteriocins like plantaricin selectively target the lipopolysaccharide layer of pathogens, inducing cytoplasmic leakage and cell death [2]. Additionally, *LP* EPS competitively blocks *Vibrio* adhesion to intestinal mucosal surfaces via molecular adsorption, forming a physical barrier. Empirical studies demonstrate that dietary supplementation with *LP* strain ND01 (isolated from seaweed) for 28 days reduced intestinal V. parahaemolyticus loads by 73%, increased survival rates by 45%, and significantly enhanced digestive enzyme (amylase and lipase) activity in infected abalone [3]. Another study on *V. alginolyticus* revealed that *LP* strain CCFM8661 upregulated defensin expression by modulating the TLR/MyD88 signaling pathway in abalone intestines, thereby strengthening innate immune responses [4]. Notably, *LP*-derived short-chain fatty acids (SCFAs) selectively promote the proliferation of beneficial bacteria (such as bifidobacteria), further suppressing *Vibrio* colonization through ecological niche competition [5]. Despite these advancements, strain-specific variability in probiotic traits—such as environmental tolerance, adhesion capacity, and antimicrobial efficacy—remains a critical challenge, necessitating systematic evaluation of antagonistic effects against abalone-specific pathogens.

In this study, four *LP* strains (NDMJ-1 to NDMJ-4) were isolated and identified from rice wine lees. Their stress resistance and safety profiles were comprehensively assessed through acid and bile salt tolerance assays, antibiotic susceptibility testing, hydrophobicity and auto-aggregation measurements, and hemolytic activity evaluation. Further in vitro antibacterial assays and abalone infection models were employed to systematically investigate the inhibitory effects of these strains and their metabolites against three pathogenic *Vibrio* species. The goal is to identify *LP* strains with potent antagonistic activity, robust environmental adaptability, and excellent biosafety, providing a scientific foundation for developing eco-friendly biocontrol agents to replace antibiotics in abalone Vibriosis management.

## 2. Materials and Methods

### 2.1. Sample Collection

Rice wine lees were purchased from a local market in Ningde City, Fujian Province, China. *Vibrio parahaemolyticus* V-25, V. alginolyticus V-3, and V. natriegens V-103 were isolated from the intestinal contents of diseased abalone (Haliotis discus hannai) collected in Pingtan, Fujian Province, and preserved at the Gut Microbiota Research Center, Institute of Animal Husbandry and Veterinary Medicine, Fujian Academy of Agricultural Sciences. *LP* strains were screened and identified from rice wine lees sourced in Ningde, Fujian.

### 2.2. Reagents and Instruments

Marine broth 2216E ((Haibo Biotechnology, Qingdao, China; HB0132-1)), MRS broth (Solarbio, Beijing, China; Cat# M8540), Bacterial Genomic DNA Extraction Kit (Tiangen Biotech, Beijing, China), nucleic acid dye, Gram staining reagents (Qingdao Haibo Biotechnology, Qingdao, China), antibiotic susceptibility disks (Hangzhou Microbial Reagent Co., Ltd., Hangzhou, China), Synergy H1 Multi-Mode Microplate Reader (BioTek Instruments, Inc., Winooski, VT, USA), BG-gdsAUTO520 Gel Imaging System (Bio-Gene Technology Ltd., Melbourne, VIC, Australia), and T100 Thermal Cycler (Bio-Rad Laboratories, Inc., Hercules, CA, USA).

### 2.3. Isolation of LP

Rice wine lees were filtered by sterile 3-ply medical gauze under a biosafety cabinet to remove coarse particles. A 1 mL aliquot was mixed with 9 mL sterile saline (0.85% NaCl) to prepare a 10-fold dilution. Serial dilutions were performed to reduce interference from non-target microbes. Diluted samples were spread onto MRS agar plates and incubated at 35 °C for 24 h. Single colonies were picked and stored in glycerol at −80 °C.

### 2.4. Strain Purification and Cultivation

Preserved isolates were inoculated into MRS broth and pre-incubated at 35 °C with shaking (100 rpm) for 4 h. Serial dilutions were streaked onto MRS agar plates and incubated at 35 °C for 24 h. Pure colonies were subcultured in MRS broth and repeatedly streaked to ensure purity.

### 2.5. Morphological Characterization

Gram staining was performed using standard protocols. Cellular morphology was observed under an optical microscope (100× oil immersion lens).

### 2.6. Physiological and Biochemical Analysis

Strain characteristics were assessed using the *Lactic Acid Bacteria* identification manual [5]. Biochemical reactions were determined using commercial microtubes (Eppendorf, Hamburg, Germany; Cat# 0030120086) according to the manufacturer’s protocols.

### 2.7. 16S rDNA Sequencing and Phylogenetic Analysis

Genomic DNA was extracted using a Tiangen Bacterial DNA Kit. The 16S rDNA gene was amplified with universal primers 27F (5′-AGA GTT TGA TCC TGG CTC AG-3′) and 1492R (5′-TAC GGC TAC CTT GTT ACG ACT T-3′). PCR conditions: 95 °C for 5 min (initial denaturation); 30 cycles of 95 °C for 30 s, 52 °C for 30 s, and 72 °C for 1.5 min; final extension at 72 °C for 5 min. Amplified products were electrophoresed on 1% agarose gels, purified, and sequenced (Sangon Biotech, Shanghai). Sequences were aligned against the NCBI database using BLAST, and a phylogenetic tree was constructed with MEGA11 (Neighbor-Joining method, 1000 bootstrap replicates). Whole-genome sequencing of NDMJ-4 was performed to confirm species identity via 16S rDNA homology analysis.

### 2.8. Probiotic Property Evaluation

#### 2.8.1. Antibacterial Activity Assay

Cell-free supernatants (CFS) from NDMJ-1 (3.5 × 10^9^ CFU/mL), NDMJ-2 (2.7 × 10^9^ CFU/mL), NDMJ-3 (8.2 × 10^8^ CFU/mL), and NDMJ-4 (4.2 × 10^9^ CFU/mL) were prepared by centrifugation (12,000× *g*, 5 min). Agar well diffusion assays were conducted on 2216E agar plates inoculated with V. parahaemolyticus V-25, V. alginolyticus V-3, or V. natriegens V-103 (10^6^ CFU/mL). Wells (6 mm diameter) were filled with 200 μL CFS and incubated at 28 °C for 12 h. Inhibition zones were measured.

#### 2.8.2. Minimum Inhibitory Concentration (MIC)

CFS was serially diluted (2-fold) in 96-well plates containing 100 μL MRS broth. Each well was inoculated with 100 μL pathogen suspension (10^6^ CFU/mL). Positive (pathogen only) and negative (medium only) controls were included. Plates were incubated at 28 °C for 24 h, and OD600 was measured (Synergy H1). MIC was defined as the lowest CFS concentration with ΔOD600 (final − initial) ≤ 0.05.

#### 2.8.3. Hemolytic Activity

Strains were streaked onto blood agar plates (BAP) and incubated at 35 °C for 24 h. Hemolysis types were classified: α-hemolysis (green/brown zones, partial lysis), β-hemolysis (clear zones, complete lysis), or γ-hemolysis (no lysis).

#### 2.8.4. Biofilm Formation Assay

Strains were cultured on Congo red-MRS agar (0.8 g/L Congo red) at 35 °C for 36 h. Biofilm-producing colonies appeared black or red, while non-producers remained pink/white.

#### 2.8.5. Antibiotic Susceptibility

Disk diffusion assays were performed using 20 antibiotics (erythromycin, minocycline, doxycycline, tetracycline, neomycin, kanamycin, gentamicin, amikacin, cefoperazone, ceftriaxone, ceftazidime, cefuroxime, cephradine, cefazolin, cephalexin, piperacillin, carbenicillin, ampicillin, oxacillin, and penicillin). Zones of inhibition were measured after 24 h incubation at 35 °C.

#### 2.8.6. Bile Salt Tolerance

Strains were inoculated into MRS broth containing 0.1%, 0.3%, or 0.5% bile salts (*w*/*v*) and incubated at 35 °C (100 rpm, 4 h). Viable counts were determined by plate enumeration.

#### 2.8.7. Acid and Alkali Resistance

Strains were exposed to MRS broth adjusted to pH 1.0, 3.0, 5.0, 7.0, or 9.0. Survival rates were quantified after 4 h incubation (35 °C, 100 rpm).

#### 2.8.8. Heat Tolerance

Strains were incubated in MRS broth at 35 °C, 45 °C, 55 °C, 65 °C, or 75 °C for 30 min. Viable cells were enumerated post-treatment.

### 2.9. Adhesion Assays

#### 2.9.1. Auto-Aggregation

Bacterial suspensions (OD600 = 0.5) were vortexed (30 s) and incubated statically at 35 °C. OD600 was measured at 1, 2, 3, and 4 h. Auto-aggregation (%) = [(OD_0_ − OD_t_)/OD_0_] × 100.

#### 2.9.2. Hydrophobicity

Bacterial suspensions (OD600 = 0.5) were mixed with xylene, chloroform, or ethyl acetate (1:1 *v*/*v*), vortexed (2 min), and phase-separated (30 min). Hydrophobicity (%) = [(OD_0_ − ODf)/OD_0_] × 100.

### 2.10. Pathogenicity Challenge in Abalone

#### 2.10.1. Abalone Rearing

Healthy abalones (19 ± 1.5 g) were obtained from a commercial farm in Xiapu, Fujian. Abalone were acclimatized in aerated seawater (salinity 3%, 18 °C) for 7 days, and then according to the Abalone Culture Conditions: stocking density ≤ 150 individuals per cubic meter of cage; water temperature is 18 °C; dissolved oxygen (DO) ≥ 6 mg/L; ammonia nitrogen < 0.1 mg/L.

#### 2.10.2. Bacterial Preparation

Newly proliferated *V. parahaemolyticus* V-25 (5 × 10^7^ CFU/mL) and *LP* NDMJ-4 (5 × 10^7^ CFU/mL) were washed twice with PBS (8000× *g*, 10 min).

#### 2.10.3. Challenge Experiment

Abalone (*n* = 20/group) were divided into Control (CK), injected with 50 μL PBS into the stomach of the abalone. Challenge (GD): injected with 50 μL *V. parahaemolyticus* (5 × 10^7^ CFU/mL). Treatment (*LP*): Challenged as GD, then immersed in *LP* NDMJ-4 suspension (5 × 10^7^ CFU/mL) for 2 h at 6 h post-infection. Mortality was recorded at 3, 6, 12, 24, 36, 48, and 72 h.

### 2.11. Statistical Analysis

Data are expressed as mean ± SD (*n* = 3). Differences were analyzed using one-way ANOVA with Duncan’s multiple range test in SPSS 22.0. Significance levels: *** *p* ≤ 0.001; ** *p* ≤ 0.01; * *p* ≤ 0.05; ns, not significant (*p* > 0.05). Graphs were generated using GraphPad Prism version 9.0.0(121).

## 3. Results

### 3.1. Isolation and Identification of NDMJ

**Figure 1:**
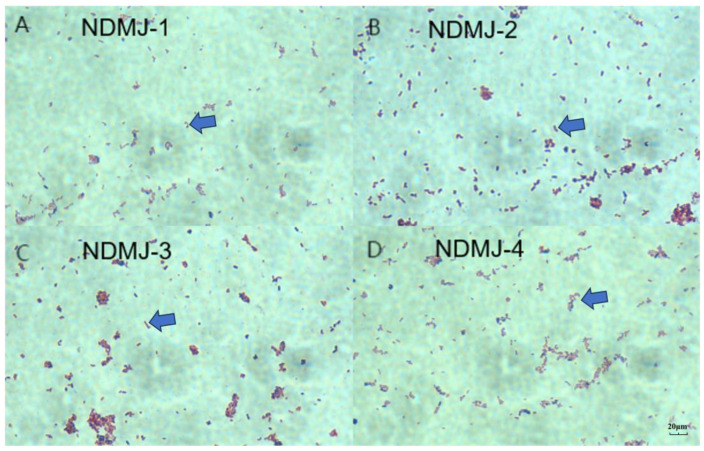
Gram staining microscopy results (100×). Note: (**A**) NDMJ-1; (**B**) NDMJ-2; (**C**) NDMJ-3; and (**D**) NDMJ-4.

Following plating of samples on MRS agar plates and subsequent isolation/purification procedures, four Lactiplantibacillus plantarum strains with high homology were selected based on 16S rDNA sequencing analysis. Subsequent Gram staining characterization revealed purple colored circular colonies with smooth surfaces and entire margins under microscopic observation. The bacterial cells exhibited typical Gram-positive rod-shaped or short bacilli morphology, as illustrated in Figure 1.

### 3.2. Identification of NDMJ Strains

**Figure 2:**
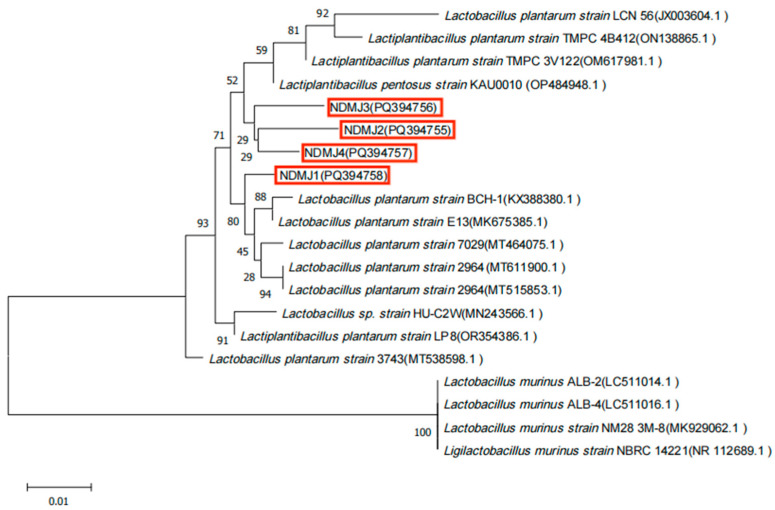
Phylogenetic analysis of isolated bacteria. Note: red box is the four *LP* isolates (NDMJ1−4).

The biochemical profiles of NDMJ-1, NDMJ-2, NDMJ-3, and NDMJ-4 strains are summarized in Table 1. All four isolates demonstrated positive reactions for esculin hydrolysis, maltose, mannitol, salicin, cellobiose, lactose, raffinose, and inulin utilization. Notably, NDMJ-1 exhibited negative sorbitol fermentation while the other three strains showed positive results. Universal bacterial primers were employed to amplify the 16S rDNA gene, yielding PCR products of approximately 1500 bp. Subsequent BLAST analysis of the sequenced fragments against the NCBI database revealed over 99% homology with *LP* reference sequences. Phylogenetic reconstruction using MEGA software (MEGAsync 5.11.1) (Figure 2) demonstrated that NDMJ-1 formed a distinct phylogenetic lineage compared to the other three isolates, while NDMJ-2 and NDMJ-4 clustered closely. Whole genome sequencing of NDMJ-4 further confirmed its taxonomic classification as *LP*, with the genomic data deposited in NCBI under accession number PRJNA1166953. Collectively, the morphological characteristics, physiological/biochemical profiles, and molecular analyses conclusively identified all four isolates as *LP.*

### 3.3. In Vitro Antibacterial Assay

**Figure 3:**
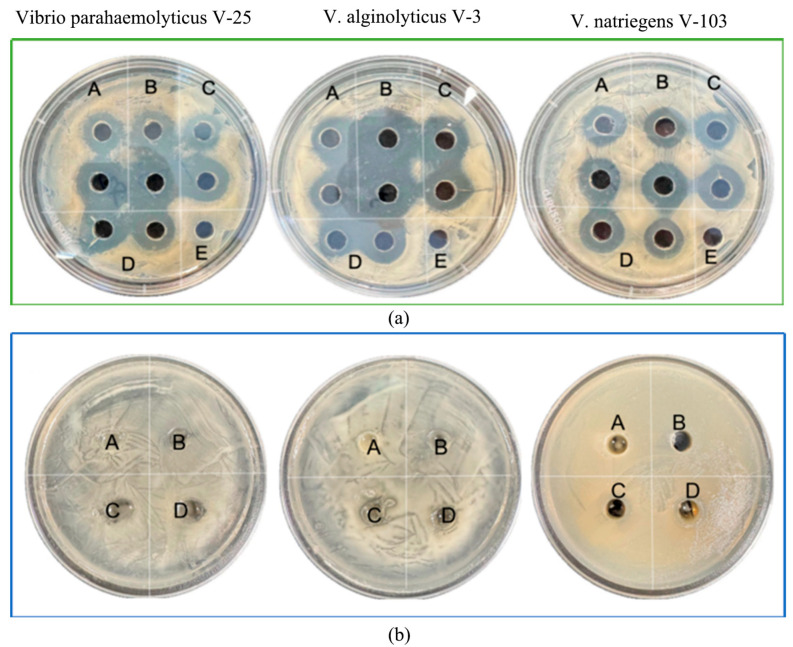
In vitro antibacterial results of isolated strains. Note: (A) NDMJ-1; (B) NDMJ-2; (C) NDMJ-3; (D) NDMJ-4; and (E) negative control. (**a**): The four *LP* isolates inhibition against three pathogenic Vibrios. (**b**): The four *LP* isolates (pH = 7) inhibition against three pathogenic Vibrios.

As illustrated in Figure 3, the four *LP* isolates (NDMJ1-4) demonstrated dose-dependent inhibition against three pathogenic Vibrios: *Vibrio parahaemolyticus V-25*, *V. alginolyticus V-3,* and *V. natriegens V-103.* Quantitative analysis revealed inhibition zone diameters of 13.0 ± 0.5 mm, 20.1 ± 0.5 mm, 18.2 ± 0.5 mm, and 13.0 ± 0.5 mm against the respective test strains (Figure 3a). To investigate the potential role of organic acids in antimicrobial activity, pH-neutralized cell-free supernatants (CFS) were prepared by adjusting the supernatant pH to 7.0. Subsequent neutralization resulted in significant attenuation (≥80% reduction) or complete elimination of inhibitory effects against all *Vibrio* strains. These findings strongly suggested that the antibacterial properties of NDMJ strains were primarily mediated by acidogenic metabolites rather than bacteriocin-like substances (Figure 3b).

### 3.4. Determination of Minimum Inhibitory Concentration (MIC) Against Vibrio Parahaemolyticus V-25

**Figure 4:**
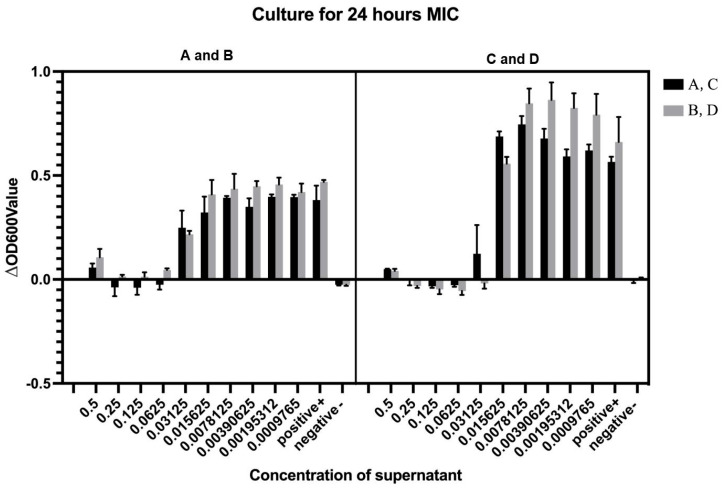
MIC minimum inhibitory concentration determination (*n* = 6). (A) NDMJ-1; (B) NDMJ-2; (C) NDMJ-3; and (D) NDMJ-4. Note: positive (*V. parahaemolyticus* V-25 only) and negative (medium only).

Cell-free supernatants (CFS) obtained from four *LP* strains by centrifugation at 8000× *g* were serially diluted for antimicrobial evaluation against *V. parahaemolyticus V-25*. Following four successive two-fold dilutions (final concentration 0.0625× original CFS), all four *LP* CFS preparations exhibited significant growth inhibition of the target pathogen. Notably, NDMJ-4 maintained inhibitory activity even after the fifth dilution cycle (0.03125×), while the other three strains (NDMJ-1, -2, and -3) showed complete loss of antimicrobial efficacy at this dilution stage. Quantitative MIC determination revealed distinct antimicrobial potency: NDMJ-1, -2, and -3 demonstrated MIC values of 0.0625× CFS concentration, whereas NDMJ-4 displayed enhanced inhibitory capacity with a lower MIC of 0.03125× CFS concentration (Figure 4).

### 3.5. Hemolytic Activity and Biofilm Formation Assessment of NDMJ Isolates

**Figure 5:**
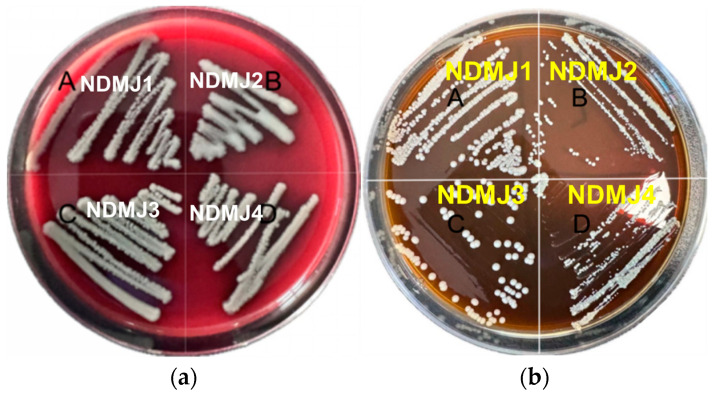
Hemolysis of isolated strains and biofilm formation ability of isolated strains. Note: (**A**) NDMJ-1; (**B**) NDMJ-2; (**C**) NDMJ-3; and (**D**) NDMJ-4. (**a**): Hemolytic evaluation conducted on blood agar plates for all NDMJ strains (NDMJ-1 to -4). (**b**): Evaluate the biofilm formation ability of four strains of all NDMJ strains (NDMJ-1 to -4).

Hemolytic evaluation conducted on blood agar plates revealed gamma-hemolytic characteristics (non-hemolytic) for all NDMJ strains (NDMJ-1 to -4), as evidenced by the development of grayish-white colonies without surrounding hemolytic zones after 24 h incubation (Figure 5a). Biofilm-forming capacity was assessed using Congo Red-supplemented MRS agar, where microbial matrix production manifests as black colony pigmentation. As shown in Figure 5b, all NDMJ isolates formed cream-colored circular colonies with opaque morphology after 24 h of cultivation, demonstrating the complete absence of Congo Red binding, indicative of biofilm-negative phenotypes.

### 3.6. Antimicrobial Susceptibility Profile of NDMJ Isolates

The four *LP* strains (NDMJ-1 to -4) were subjected to standardized antimicrobial susceptibility testing against 20 clinically relevant antibiotics, as detailed in Table 2. All isolates demonstrated universal susceptibility to second/third-generation cephalosporins (cefuroxime and cefotaxime), with notable resistance to first-generation cephalexin. Complete resistance patterns were observed against tetracyclines (tetracycline), aminoglycosides (neomycin and kanamycin), and β-lactams (oxacillin and cephalexin). Conversely, maintained susceptibility was recorded for extended-spectrum penicillins (piperacillin, carbenicillin, and ampicillin) and selected cephalosporins. This resistance profile aligns with typical intrinsic resistance patterns of *LP* [6], suggesting potential probiotic safety in clinical applications.

### 3.7. Stress Tolerance of NDMJ Isolates

#### 3.7.1. Bile Salt Tolerance

The bile salt resistance profiles of NDMJ-1 to -4 strains are presented in Table 3. Quantitative analysis revealed a concentration-dependent survival pattern, where viable cell counts exhibited significant reduction (*p* < 0.05) with increasing bile salt concentrations (0.1–0.3% *w*/*v*). Notably, all four *LP* isolates maintained measurable viability (10^6^ CFU/mL) at the critical intestinal bile concentration of 0.3%, demonstrating substantial bile salt tolerance. This survival threshold exceeds the minimum requirement (10^5^ CFU/mL) for probiotic microorganisms in gastrointestinal transit, suggesting potential for intestinal colonization and probiotic functionality.

#### 3.7.2. Acid Tolerance

The pH tolerance profiles of NDMJ-1 to -4 strains are detailed in Table 4. Following 4 h exposure to neutral (pH 7.0) and alkaline (pH 8.0) conditions, viable cell counts maintained stability with titers exceeding 5.78 × 10^4^ CFU/mL as determined by plate enumeration. Remarkably, under acidic challenge (pH 3.0), all *LP* isolates retained substantial viability (>5.43 × 10^4^ CFU/mL), exhibiting < 1-log reduction compared to neutral conditions. This survival rate surpasses the critical threshold (10^3^ CFU/mL) for probiotic persistence in gastric environments, demonstrating exceptional acid tolerance compatible with human gastrointestinal transit requirements.

#### 3.7.3. Thermal Tolerance

Thermal resistance evaluation of the four *LP* strains revealed temperature-dependent survival patterns as detailed in Table 5. Following 30 min thermal challenges, all isolates maintained cultivability at mesophilic (35 °C) and sub-lethal (55 °C) conditions via plate enumeration, though with significant viability reduction (>2-log decrease at 55 °C versus 35 °C control). Complete thermal inactivation occurred at 75 °C exposure, with no detectable colonies post-treatment. These findings delineate an optimal growth range (30–45 °C, peaking at 35 °C) and critical thermal limits (<60 °C) characteristic of *mesophilic lactobacilli*. The observed thermosensitivity profile suggests potential for food processing applications requiring moderate heat resistance, while confirming susceptibility to standard pasteurization protocols.

### 3.8. Adhesion-Related Properties of NDMJ Isolates

#### 3.8.1. Auto-Aggregation Capacity

**Figure 6:**
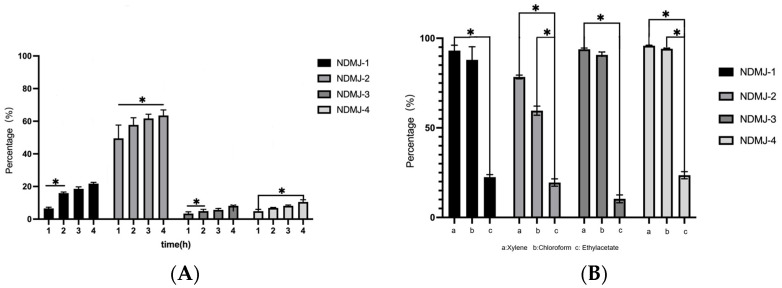
Auto-aggregation and Hydrophobicity of NDMJ-1, NDMJ-2, NDMJ-3, and NDMJ-4. (**A**) Auto-aggregation. (**B**) Hydrophobicity. Note: 1: xylene; 2: Chloroform; 3: Ethyl acetate. *: Significant differences (*p* < 0.05).

As shown in Figure 6, all four *LP* strains exhibited time-dependent auto-aggregation over 4 h. NDMJ-2 demonstrated superior auto-aggregation capacity, increasing from 55.29% to 65.94%, significantly higher than the other strains (*p* < 0.05). NDMJ-1 showed moderate aggregation (6.05% → 22.34%), while NDMJ-3 and NDMJ-4 exhibited limited aggregation (2.66% → 7.77% and 5.67% → 11.47%, respectively). Statistical analysis revealed significant temporal differences: NDMJ-1 at 2 h, NDMJ-3 at 1–2 h, and NDMJ-4 at 1–4 h (*p* < 0.05), with no significant variations at other timepoints (*p* > 0.05).

#### 3.8.2. Surface Hydrophobicity

Hydrophobic interactions analyzed through microbial adhesion to solvents (MATS) are presented in Figure 6. All isolates exhibited strong affinity for non-polar xylene (>94.31% hydrophobicity), with NDMJ-1 and NDMJ-4 exceeding 95%. In polar solvents, hydrophobicity decreased progressively: ethyl acetate (moderate) > chloroform (lowest). Notably, NDMJ-4 maintained relatively higher chloroform tolerance compared to other strains, while NDMJ-3 showed elevated ethyl acetate affinity. Significant inter-solvent differences were observed between xylene and ethyl acetate across all strains (*p* < 0.05). These hydrophobic profiles correlate with bacterial surface protein composition, suggesting strain-specific adhesion mechanisms critical for probiotic-host interactions.

### 3.9. Vibrio Parahaemolyticus Challenge Assay

**Figure 7:**
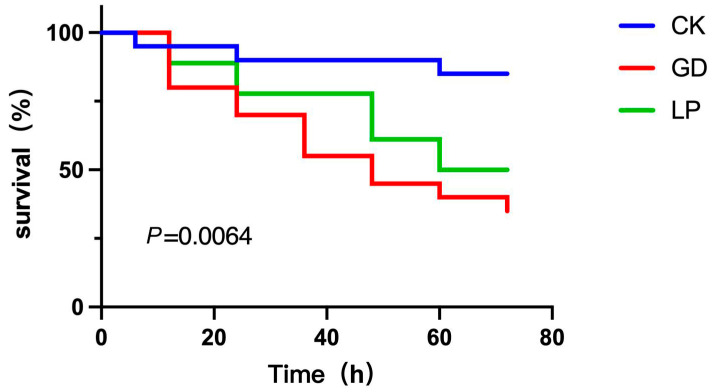
Abalone mortality plots of three different treatment groups. Note: CK: control group; GD: challenge group; *LP*: treatment group.

As shown in Figure 7, all four *LP* strains exhibited time-dependent auto-aggregation abalone specimens infected with *V. parahaemolyticus* V-25 exhibited characteristic pathogenicity symptoms, including lethargy and anorexia within 6 h post-infection. Cumulative mortality progressed significantly (*p* < 0.05) from 6 h to 72 h, with survival rates at 72 h post-challenge quantified as follows (Figure 7): sterile control (PBS-injected): 85% viability; challenge control (V-25 only): 35% survival; therapeutic group (V-25 + NDMJ-4): 50% survival. Therapeutic intervention with NDMJ-4 significantly enhanced survival rates compared to the challenge control (Δ15%, *p* < 0.05). Notably, the sterile control group displayed unexpected 15% mortality, potentially attributable to PBS-induced stress responses in Haliotis discus physiology. These results demonstrate that NDMJ-4 supplementation confers protective efficacy against *V. parahaemolyticus* infection in abalone, reducing mortality by 42.9% relative to untreated infected specimens, over 4 h. NDMJ-2 demonstrated superior auto-aggregation capacity, increasing from 55.29% to 65.94%, significantly higher than the other strains (*p* < 0.05). NDMJ-1 showed moderate aggregation (6.05% → 22.34%), while NDMJ-3 and NDMJ-4 exhibited limited aggregation (2.66% → 7.77% and 5.67% → 11.47%, respectively). Statistical analysis revealed significant temporal differences: NDMJ-1 at 2 h, NDMJ-3 at 1–2 h, and NDMJ-4 at 1–4 h (*p* < 0.05), with no significant variations at other timepoints (*p* > 0.05).

## 4. Discussion

The emergence of *Vibrio* pathogens, particularly *Vibrio parahaemolyticus*, poses a persistent threat to marine aquaculture, necessitating innovative biocontrol strategies. These Gram-negative bacteria exploit multiple infection routes, ranging from direct mucosal colonization to biofilm-mediated persistence, thereby compromising abalone health and destabilizing global aquaculture economies [7,8,9]. In this context, *LP* stands out as a multifaceted probiotic candidate. Its evolutionary adaptation to diverse niches, along with its Generally Recognized As Safe (GRAS) status, uniquely positions it for marine applications [10]. Our isolation of four *LP* strains (NDMJ-1 to -4) from traditional Ningde rice wine lees not only highlights the untapped microbial diversity of artisanal fermentation systems but also uncovers strains with a certain potential against Vibriosis, thereby bridging traditional food microbiology with contemporary aquaculture needs.

The oral delivery paradigm imposes stringent selection pressures on probiotics. Beyond mere survival, successful strains must sustain metabolic activity across the gastrointestinal tract’s physicochemical gradients. Our data reveal that all NDMJ strains exhibit remarkable aciduricity, maintaining viability (>10^6^ CFU/mL) at pH 3.0—a trait mediated by proton-pumping F_1_F_0_-ATPases and stress-responsive chaperones, as characterized in model *lactobacilli* [11]. Notably, the observed <1-log reduction under gastric-mimetic conditions surpasses the FAO/WHO-recommended threshold (10^5^ CFU/mL) for probiotic efficacy, suggesting robust enteric survival [12].

Bile salt resistance is another critical determinant that involves complex adaptations, including membrane lipid remodeling through bile salt hydrolase (BSH) activity, upregulation of efflux pumps, and mitigation of oxidative stress via glutathione cycling [13,14]. The sustained growth of NDMJ strains in the presence of 0.3% bile salts—a concentration that mimics the porcine jejunum—indicates an evolutionary optimization for intestinal persistence. This observation aligns with recent metagenomic insights that demonstrate the dominance of *LP* in high-bile environments, mediated by plasmid-encoded resistance clusters [15].

The mesophilic growth range (35–45 °C) observed in NDMJ strains reflects ecological adaptation to host-associated niches rather than environmental persistence. The sharp viability decline above 55 °C, attributed to ribosomal thermolability and membrane lipid phase transitions, presents both challenges and opportunities. While necessitating cold-chain maintenance for probiotic formulations, this thermal sensitivity ensures biocontainment: a critical feature for preventing environmental persistence of supplemented strains [16].

Auto-aggregation and hydrophobicity metrics provide critical insights into the host-microbe cross-talk. The superior auto-aggregation of NDMJ-2 (65.94%) correlates with surface-exposed aggregation-promoting factors (APFs), which are typically fibronectin-binding proteins that mediate both intra-species cohesion and mucosal adherence [17]. The >94% xylene hydrophobicity observed across strains suggests the dominance of surface-layer (S-layer) proteins with hydrophobic domains, a trait that has been co-opted from ancestral gut colonization strategies. Such hydrophobic interactions likely facilitate the competitive exclusion of *Vibrio* pathogens by occupying mucosal binding sites, a mechanism that has been observed in coral probiotic systems [18]. The γ-hemolytic phenotype and biofilm-negative status of NDMJ strains address two critical biosafety concerns. The absence of hemolysin production eliminates the risks of erythrocyte lysis, which is a notorious side effect of some commercial probiotics [19,20,21]. Concurrently, the lack of biofilm formation mitigates industrial fouling risks while aligning with the FDA’s stringent regulations on food-grade microbes [22,23,24].

The antibiotic susceptibility profiles further validate the safety of *Lactobacillus* strains. The observed sensitivity to β-lactams and aminoglycosides, despite the intrinsic resistance of *Lactobacillus* to vancomycin, minimizes the risks associated with horizontal gene transfer, which is a growing concern in aquaculture settings [25]. This profile aligns with the World Organization for Animal Health’s (WOAH) One Health framework, which advocates for the use of probiotics as mitigators of antimicrobial resistance (AMR) rather than contributors [26,27].

The 42.9% reduction in mortality observed in NDMJ-4-supplemented abalone (compared to 70% in controls) mirrors significant advancements in probiotic research involving finfish, where *Lactobacillus* spp. disrupt *Vibrio* quorum sensing through AI-2 mimicry [28]. The specificity of this effect is evidenced by the unchanged mortality rates in sterile controls, which excludes nonspecific immune stimulation and suggests direct inhibition of pathogens. The observed reduction in mortality conferred by *LP* against Vibrio infection, as compared to PBS and challenge controls, arises from a multifaceted interplay of bacterial antagonism and host immunomodulation. As a Gram-positive probiotic, *LP* directly compromises the structural integrity of Gram-negative Vibrio through secreted organic acids (notably lactic acid) that destabilize lipopolysaccharide (LPS) layers in the outer membrane, alongside bacteriocins capable of pore formation—collectively disrupting virulence gene expression (ctxA and tcpA) and suppressing pathogen viability [29,30]. Concurrently, *LP* fortifies host defenses by enhancing intestinal barrier function via tight junction protein upregulation and orchestrating immune responses that skew toward anti-inflammatory cytokine production (IL-10) while dampening pro-inflammatory cascades (TNF-α, IL-8) [31]. This dual capacity to directly target Gram-negative pathogens despite phylogenetic divergence, while simultaneously reinforcing host mucosal immunity, underscores the unique potential of *LP* as a broad-spectrum therapeutic agent against enteric infections. Furthermore, metabolomic studies on related strains have identified secreted cyclic dipeptides that destabilize *Vibrio* membranes, a plausible mechanism that warrants further investigation [32].

In this study, we mainly adopted the method of immersing NDMJ supernatant because the immersion administration of NDMJ for treating abalone Vibriosis offers comprehensive advantages over oral or feed-based delivery. This method allows the probiotics to directly penetrate the abalone’s body surface, gills, and digestive tract via the water column, simultaneously eliminating both internal and external *Vibrio* pathogens [33]. It is particularly effective during the feeding cessation period in diseased abalone, overcoming the failure risk associated with oral methods that rely on active ingestion. Its gentle, non-contact nature avoids mechanical injury from forced feeding and intestinal stress, while the probiotics secreted antimicrobial substances into the environment, competitively inhibit *Vibrio* biofilm formation, and improve water quality—thereby blocking the infection chain at its source [34]. Furthermore, the bacteria can rapidly target the hepatopancreas via the gills to directly neutralize toxins and activate immune repair, whereas the oral route suffers from low efficiency due to compromised intestinal barriers. Operationally, immersion enables efficient group-level treatment and ensures probiotic viability, significantly outperforming the viability losses inherent in feed processing and digestion. This establishes a comprehensive triple-action control mechanism: environmental disinfection, surface colonization suppression, and organ repair [35,36].

While this study demonstrates the promising probiotic potential of *LP* NDMJ-4 against *Vibrio* pathogens in abalone, several limitations warrant consideration. First, the in vitro antibacterial assessment relied exclusively on cell-free supernatants, leaving the direct antagonistic mechanisms of viable probiotics unexplored. Second, the in vivo validation was confined to a single strain (NDMJ-4) and one pathogen (*V. parahaemolyticus*), necessitating expanded trials with other NDMJ strains and prevalent *Vibrio* species (*V. alginolyticus*). Future work should focus on the following: (i) elucidating the specific antimicrobial metabolites and their modes of action; (ii) optimizing viable probiotic delivery methods (encapsulation) to enhance thermostability and gastrointestinal survival; (iii) evaluating long-term field efficacy and ecological impacts in diverse aquaculture systems; and (iv) exploring synergistic effects with other biocontrol agents to develop multi-targeted strategies.

## 5. Conclusions

This study characterized four *LP* strains (NDMJ-1 to -4) isolated from Ningde rice wine lees, demonstrating robust stress tolerance (acid, bile, and thermal), adhesion competency, and antibiotic sensitivity compliant with aquaculture probiotics standards. Notably, NDMJ-4 exhibited superior *vibrio*-inhibitory activity, reducing *V. parahaemolyticus*-induced abalone mortality by 42.9% in challenge assays. These findings underscore NDMJ-4’s potential as a sustainable alternative to chemical therapeutics in abalone farming, thereby warranting further investigation into its antimicrobial mechanisms and large-scale application protocols.

## Figures and Tables

**Table 1:** Biochemical characteristics of the isolates’ strain.

Item	NDMJ-1	NDMJ-2	NDMJ-3	NDMJ-4
Esculin	+	+	+	+
Maltose	+	+	+	+
Salicin fermentation tube	+	+	+	+
Sorbitol fermentation tube	−	+	+	+
Cellobiose	+	+	+	+
Lactose	+	+	+	+
Raffinose	+	+	+	+
Sucrose	+	+	+	+
Inulin	+	+	+	+

Note: +, positive; −, negative.

**Table 2:** Sensitivity test of isolated strains to different drugs.

Antibiotic Class	Drug Content (μg/Tablet)	Probiotics No.			
		NDMJ-1	NDMJ-2	NDMJ-3	NDMJ-4
β-Lactams	Erythromycin (15)	R	S	S	S
	Minocycline (30)	R	S	S	R
	Doxycycline (30)	S	R	R	R
	Tetracycline (30)	R	R	R	R
	Neomycin (30)	R	R	R	R
	Kanamycin (30)	R	R	R	R
	Gentamycin (10)	S	S	R	R
	Amikacin (30)	R	S	S	S
	Cefoperazone (75)	R	S	S	S
	Ceftriaxone (30)	R	R	S	S
	Ceftazidime (30)	S	S	R	S
	Cefuroxime (30)	S	S	S	S
Aminoglycosides	Cefuroxime (30)	R	S	S	S
	Cefazolin (30)	S	S	R	S
	Cephalexin (30)	R	R	R	R
	Piperacillin (100)	S	S	S	S
Macrolides	Carbenicillin (100)	S	S	S	S
Tetracyclines	Ampicillin (10)	S	S	S	S
	Oxacillin (1)	R	R	R	R
	Penicillin (10)	R	R	S	S

Note: (S) sensitive, (R) resistant.

**Table 3:** Tolerance of the isolated strains under different concentrations of bile salts.

Treat 4 h	Live Bacteria Concentration (CFU/mL) at Different Bile Salt Contents			
	Initial Concentration (0.0%)	0.1%	0.3%	0.5%
NDMJ-1	4.36 × 10^4^ ± 3.27 × 10^2^	3.29 × 10^4^ ± 1.00 × 10^2^	1.97 × 10^4^ ± 1.53 × 10^2^	1.26 × 10^4^ ± 1.00 × 10^2^
NDMJ-2	4.80 × 10^4^ ± 1.53 × 10^2^	5.30 × 10^4^ ± 1.00 × 10^2^	1.27 × 10^3^ ± 2.50 × 10^1^	8.40 × 10^3^ ± 5.00 × 10^1^
NDMJ-3	4.80 × 10^4^ ± 1.53 × 10^2^	4.21 × 10^4^ ± 1.00 × 10^2^	8.70 × 10^3^ ± 5.00 × 10^1^	7.00 × 10^3^ ± 5.00 × 10^1^
NDMJ-4	5.11 × 10^4^ ± 3.61 × 10^2^	4.42 × 10^4^ ± 2.50 × 10^2^	2.78 × 10^4^ ± 2.00 × 10^2^	1.35 × 10^4^ ± 1.00 × 10^2^

**Table 4:** Tolerance of the isolated strains under different acidity.

Processing 4 h	Live Bacteria Concentration at Different pH (CFU/mL)					
	(pH = 6.5)	pH = 1.0	pH = 3.0	pH = 5.0	pH = 7.0	pH = 9.0
NDMJ-1	7.07 × 10^5^ ± 1.55 × 10^4^	3.51 × 10^3^ ± 9.00 × 10^1^	8.50 × 10^4^ ± 9.50 × 10^2^	5.20 × 10^5^ ± 1.00 × 10^2^	8.03 × 10^5^ ± 6.24 × 10^3^	4.20 × 10^4^ ± 1.00 × 10^3^
NDMJ-2	4.90 × 10^6^ ± 1.00 × 10^5^	4.10 × 10^4^ ± 1.00 × 10^2^	5.51 × 10^5^ ± 8.54 × 10^3^	1.05 × 10^6^ ± 5.00 × 10^2^	7.10 × 10^6^ ± 1.00 × 10^3^	7.70 × 10^5^ ± 1.00 × 10^2^
NDMJ-3	6.33 × 10^5^ ± 1.55 × 10^4^	7.90 × 10^3^ ± 9.00 × 10^1^	4.62 × 10^4^ ± 7.23 × 10^2^	3.20 × 10^5^ ± 1.00× 10^2^	4.60 × 10^5^ ± 1.00 × 10^2^	1.15 × 10^5^ ± 5.00 × 10^2^
NDMJ-4	3.10 × 10^6^ ± 1.00 × 10^5^	5.10 × 10^3^ ± 1.00 × 10^2^	5.61 × 10^4^ ± 8.08 × 10^2^	9.10 × 10^4^ ± 1.00 × 10^2^	2.05 × 10^6^ ± 5.00 × 10^4^	1.05 × 10^6^ ± 5.00 × 103

**Table 5:** Tolerance of the isolated strains under different temperatures.

Treat 30 min	Live Bacteria Concentration at Different Temperatures (CFU/mL)				
	35 °C	45 °C	55 °C	65 °C	75 °C
NDMJ-1	2.41 × 10^5^ ± 3.61 × 10^3^	2.50 × 10^5^ ± 2.00 × 10^3^	4.12 × 10^2^ ± 7.64 × 10^0^	0 ± 0	0 ± 0
NDMJ-2	4.37 × 10^6^ ± 3.61 × 10^2^	4.41 × 10^6^ ± 1.53 × 10^2^	3.45 × 10^3^ ± 5.00 × 10^1^	5.00 × 10^1^ ± 1.00 × 10^0^	0 ± 0
NDMJ-3	2.08 × 10^6^ ± 1.53 × 10^2^	1.92 × 10^6^ ± 1.00 × 10^2^	4.50 × 10^2^ ± 1.00 × 10^1^	0 ± 0	0 ± 0
NDMJ-4	1.86 × 10^6^ ± 1.00 × 10^2^	1.62 × 10^6^ ± 1.00 × 10^2^	3.12 × 10^2^ ± 7.64 × 10^0^	2.00 × 10^1^ ± 1.00 × 10^0^	0 ± 0

## Data Availability

The original contributions presented in this study are included in the article. Further inquiries can be directed to the corresponding author.
